# Epigenetic Marks and Variation of Sequence-Based Information Along Genomic Regions Are Predictive of Recombination Hot/Cold Spots in *Saccharomyces cerevisiae*

**DOI:** 10.3389/fgene.2021.705038

**Published:** 2021-06-29

**Authors:** Guoqing Liu, Shuangjian Song, Qiguo Zhang, Biyu Dong, Yu Sun, Guojun Liu, Xiujuan Zhao

**Affiliations:** ^1^School of Life Sciences and Technology, Inner Mongolia University of Science and Technology, Baotou, China; ^2^Inner Mongolia Key Laboratory of Functional Genomics and Bioinformatics, Inner Mongolia University of Science and Technology, Baotou, China; ^3^School of Life Sciences, Inner Mongolia University, Hohhot, China

**Keywords:** recombination hotspots, DNA physical property, classifier, epigenetic mark, optimal feature set

## Abstract

Characterization and identification of recombination hotspots provide important insights into the mechanism of recombination and genome evolution. In contrast with existing sequence-based models for predicting recombination hotspots which were defined in a ORF-based manner, here, we first defined recombination hot/cold spots based on public high-resolution Spo11-oligo-seq data, then characterized them in terms of DNA sequence and epigenetic marks, and finally presented classifiers to identify hotspots. We found that, in addition to some previously discovered DNA-based features like GC-skew, recombination hotspots in yeast can also be characterized by some remarkable features associated with DNA physical properties and shape. More importantly, by using DNA-based features and several epigenetic marks, we built several classifiers to discriminate hotspots from coldspots, and found that SVM classifier performs the best with an accuracy of ∼92%, which is also the highest among the models in comparison. Feature importance analysis combined with prediction results show that epigenetic marks and variation of sequence-based features along the hotspots contribute dominantly to hotspot identification. By using incremental feature selection method, an optimal feature subset that consists of much less features was obtained without sacrificing prediction accuracy.

## Introduction

Meiotic recombination is crucial to gametogenesis as it helps the faithful separation of homologous chromosomes into gametes by forming chiasma (Coop and Przeworski, 2007). Abnormal or no recombination between homologous chromosomes would cause aneuploidy in gametes and affect health in offspring. For example, 10–30% of zygotes are aneuploid and approximately 30% of maternally derived cases with chromosome mis-segregation are associated with failure of crossover formation (MacLennan et al., 2015). Recombination also attracts researchers’ attention because it drives genome evolution by producing genetic diversity (Webster and Hurst, 2012).

During meiosis, DNA double-strand break initiates recombination at leptotene stage of first round of meiotic division (MI) (Baudat et al., 2013). Only a few of DSB sites across a chromosome are selected to designate cross-over (CO) that is followed by CO maturation (Wang et al., 2017). DSB hot sites are strongly correlated with recombination rate, and hence are used to indicate recombination hotspots. In contrast with hotspots, coldspots refer to the genomic regions undergo no or extremely low level of recombination. Recombination rate is unevenly distributed along chromosomes, but it is still unclear that how hotspots are arranged across the genome. DNA sequence features like PRDM9-binding motif (Myers et al., 2008), GC content (Galtier et al., 2001), GC-skew (Smagulova et al., 2011), SNP pattern (Pratto et al., 2014), and dinucleotide bias (Liu and Li, 2008) were known to correlate recombination rate, but the effects of DNA physical properties and DNA shape on recombination need further investigation.

Computational identification of recombination hotspots may help people get quick information about recombination and relieve the time-consuming experimental determination of hotspots with high cost. As reviewed in Yang et al., 2020, there are some existing models for hotspot identification at present (Zhou et al., 2006; Jiang et al., 2007; Liu et al., 2012, 2017; Chen et al., 2013; Li et al., 2014; Qiu and Xiao, 2014; Jani et al., 2018; Zhang and Kong, 2019; Khan et al., 2020). Almost all of the models were DNA sequence dependent and epigenetic marks that have been increasingly freely available were not considered. For example, nucleosome depletion (Pan et al., 2011) and H3K4me3 mark (Borde et al., 2009) were not considered in the models. Although in our previous study, we attempted to include the effect of nucleosome occupancy (Zhang and Liu, 2014), the use of MNase-seq data derived from non-meiotic cells may not provide reliable information. In fact there are more and more chromatin level factors and DNA-protein binding have been shown to affect recombination (Getun et al., 2010; Zhang et al., 2011; Cesarini et al., 2012; de Castro et al., 2012; Sommermeyer et al., 2013; Yamada et al., 2013; Gittens et al., 2019; Pyatnitskaya et al., 2019; Heldrich et al., 2020; Karányi et al., 2020; Paiano et al., 2020; Serrano-Quílez et al., 2020). In addition, DNA shape and physical properties were also shown to affect recombination hotspot identification (Chen et al., 2013), but the importance of individual DNA shape feature is unclear because they were implicitly incorporated in the model in the form of pseudo nucleotide composition. Furthermore, as far as we know, DNA shape parameter sets derived from different groups differ a lot (Liu et al., 2016), suggesting that the accuracy of the parameter estimation is unclear. In this aspect, it is also worth noting that the DNA shape parameters are sequence context-dependent (Zhou et al., 2013), and context-dependent estimation of DNA shape parameters may assist hotspot prediction. Indeed, DNA shape features were used in the prediction of DSB sites (not meiotic DSB sites) in human cell lines (Mourad et al., 2018).

In this study, we first characterized the recombination hot/cold spots with regard to DNA sequence-based features and some other features like histone modification and Top2 binding signal, and then developed several classifiers to discriminate recombination hotspots from coldspots. Comparison with other models demonstrated the good performance of our model.

## Materials and Methods

### Benchmark Datasets

**FIGURE 1:**
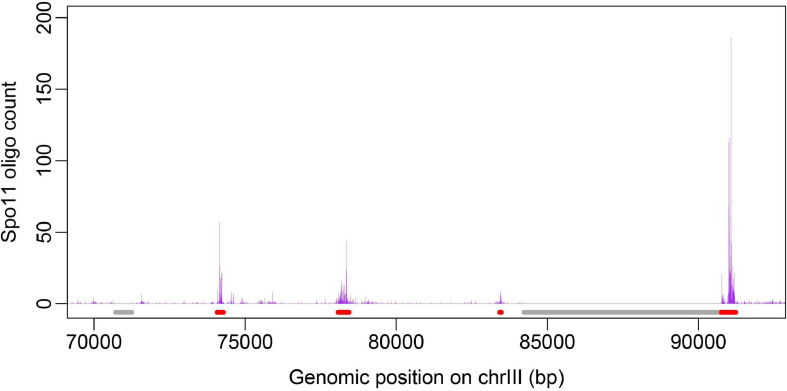
Distribution of hot/cold spots along chromosome is shown with Spo11-oligo signal taken from Pan et al. (2011). Genomic regions marked in red denote hotspots and gray represent coldspots defined in this study.

Benchmark datasets here include two datasets: positive and negative dataset. Positive dataset consists of 3,600 recombination hotspots defined by other group based on high-resolution Spo11-oligo sequencing data (Pan et al., 2011). Generally speaking, the construction of negative dataset is much trickier than positive one in binary classification, because the negative samples are much more enriched than positive samples, leading to unbalance between positive and negative dataset. Moreover, negative samples selected to represent non-positive samples may include a big noise. For example, there is a tremendous number of “non-hotspot” regions in the genome, but recombination rate at those regions are not necessarily low because they are just undetected by peak calling algorithm for hotspot identification. To address this problem, we defined negative dataset of recombination coldspots as the genomic regions of at least 500 bp long with no Spo11-oligo signal (zero value) based on the full Spo11-oligo map (Pan et al., 2011). Defining coldspots in this way, we focus on relatively large cold regions with low recombination, which may not result from the noise or limited sequencing depth in Spo11-oligo seq. To give a visual inspection, a plot of hot/cold spot regions along with Spo11-oligo signal is shown (Figure 1). The final benchmark consists of 3,600 hotspots and 2,538 coldspots. The length distribution of the hot/cold spots sequences was provided in Supplementary Information (Supplementary Figure 1). All datasets used in this study were provided in Supplementary data (Supplementary Table 1).

It should be highlighted that the hotspots and coldspots used in this study are not defined as in previous models in ORF-based way (Zhou et al., 2006; Jiang et al., 2007; Liu et al., 2012, 2017; Chen et al., 2013; Li et al., 2014; Qiu and Xiao, 2014; Jani et al., 2018; Zhang and Kong, 2019; Khan et al., 2020), but are based on the high-resolution Spo11-oligo seq data. In this way we train our models on “true” hotspots, rather than on hot/cold ORFs that are not necessarily equivalent to “true” hotspots.

### Feature Extraction

Three types of features are used in our prediction (Table 1): sequence compositional information, DNA physical properties and non-DNA features. Features that indicates sequence compositional information includes: GC content, GC-skew, mutual information and k-mer composition. Features used to reflect DNA physical properties include DNA shape parameters (Zhou et al., 2013), DNA rigidity, etc. Non-DNA features we used include some epigenetic marks (H3K4me3 and H3K56ac), MNase-seq signal, and Top2 binding signal. These features are calculated in the following way.

**TABLE 1:** Features used in this study.

Feature type	Features	Feature extraction manner	Feature number (96 + 4^*k*^)	References
DNA composition	GC content	Mean + var	2	–
	GC-skew	Mean + var	2	–
	MI	Mean + var	2	Luo et al., 1998
	K-mer composition	Overall	4^*k*^	–
DNA shape	MGW, HelT, rise, roll, shift, slide, tilt, buckle, opening, ProT, shear, stagger, and stretch	Mean + var	13 × 2	Zhou et al., 2013
DNA physical properties	EP	Mean + var	2	Zhou et al., 2013
	Rigidity	Mean + var	2	Scipioni et al., 2002
	Gibbs free energy	Mean + var	2	Ignatova et al., 2008
	Enthalpy	Mean + var	2	Ignatova et al., 2008
	Entropy	Mean + var	2	Ignatova et al., 2008
	Parameter set (Chen)	Mean + var	12 × 2	Chen et al., 2012
	Parameter set (Liu)	Mean + var	12 × 2	Liu et al., 2021
Non-DNA features	H3K4me3 (GSE11004)	Mean	1	Borde et al., 2009
	H3K56ac (GSE37487)	Mean	1	Karányi et al., 2020
	H3K4me3 (GSE59005)	Mean	1	Hu et al., 2015
	H3K56ac (GSE59005)	Mean	1	Hu et al., 2015
	MNase-seq (GSE59005)	Mean	1	Hu et al., 2015
	Top2-CC-seq (GSE136675)	Mean	1	Gittens et al., 2019

*MGW, minor groove width; ProT, propeller twist; HelT, helical twist; EP, electrostatic potential; Parameter set (Chen) include 12 features collected in Chen et al. (2012); Parameter set (Liu) include force constants and equilibrium structure parameters for 10 unique dinucleotides presented in Liu et al. (2021). Data of Top2 CC-seq used here refers to the processed data of VP16-treated sample (RA7-RA13_Cer3H4L2_MJ551_pdr1mre11_VP16.FullMap); H3K4me3, H3K56ac, and MNase-seq data were derived from meiotic cells at 4 h during sporulation when recombination initiates. For some non-DNA features, data resolution is not high enough (e.g., H3K4me3_GSE11004), which would impede us to obtain reliable high-resolution variation patterns of the features at hot/cold spots. Therefore, variances of non-DNA features along hot/cold spots were not considered.*

(1) $$p_{t}\left(k\right)=\left\{ \begin{array}{c} \frac{N_{t}}{\sum_{t=1}^{4^{k}}N_{t}}\;k=1,2\\ \frac{N_{t}+1}{\sum_{t=1}^{4^{k}}N_{t}+4^{k}}\;k=3,4,5,6 \end{array}\right.$$

(2) $$GC-content=\frac{N_{G}+N_{C}}{N_{A}+N_{T}+N_{G}+N_{C}}$$

(3) $$GC-skew=\frac{N_{G}-N_{C}}{N_{G}+N_{C}}$$

(4) $$MI=\sum_{i,j}p_{ij}\log_{2}\frac{p_{ij}}{p_{i}p_{j}}$$

where *N*_*i*_ represents the occurrence number of nucleotide *i* in a DNA sequence; *p*_*i*_ or *p*_*j*_ (*i*,*j* = *A*,*G*,*C*,*T*) is the fraction of nucleotide *i* or *j* and *p*_*ij*_ is the fraction of dinucleotide *ij* in a sequence. Mutual information (*MI*) describes the overall deviation of observed probabilities of dinucleotides from those expected from mononucleotide probabilities (Luo et al., 1998). *p*_*t*_(*k*)represents the composition of *t-*th k-mer (oligonucleotide of k bp in length) in a sequence, which refers to the occurrence probability of the k-mer counted by a sliding step of 1 bp along the sequence. To avoid the shortcoming caused by small sequence length in the calculation of k-mer compositional probability, Laplacian correction was done for k-mers where *k* > 2 [see eq. (1)].

DNA shape parameters were calculated at base pair step resolution using R package DNAshapeR (Zhou et al., 2013). With respect to DNA physical property, we also used the parameter set collected in a previous study (Chen et al., 2012), three DNA thermodynamic property parameters including Gibbs free energy, entropy and enthalpy (Ignatova et al., 2008), DNA rigidity (Scipioni et al., 2002; Liu et al., 2018), and parameter set including equilibrium base-pair step parameters (Supplementary Figure 2) and force constants which were estimated in our previous study by using crystal structure of protein-DNA complexes (Liu et al., 2019, 2021). The values of the parameters were listed in Supplementary Tables 2–4.

**FIGURE 2:**
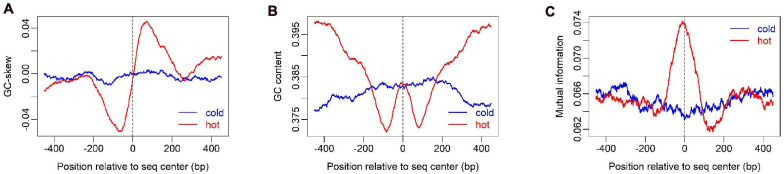
Sequence compositional feature profile of hot/cold spots. GC-skew **(A)**, GC-content **(B)**, and Mutual information **(C)** were calculated along sequences by using a sliding window of 100 bp, 100 bp, and 200 bp respectively.

**FIGURE 3:**
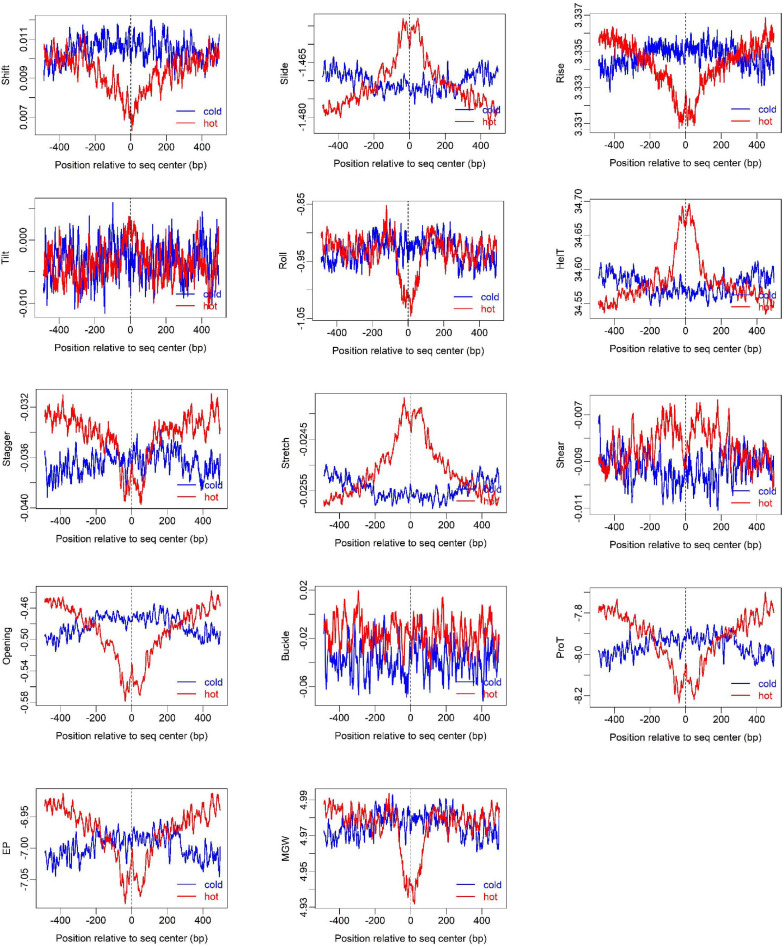
Distribution of DNA shape and physical properties at hot/cold spots. The plots were smoothed with a 10-bp moving average.

**FIGURE 4:**
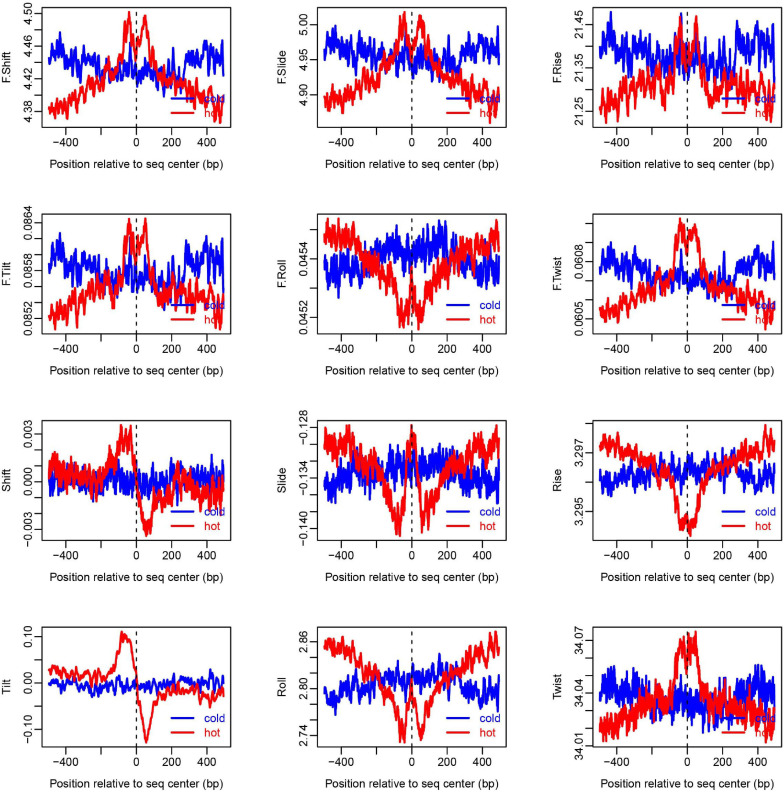
Distribution of DNA base-pair-step parameters at hot/cold spots. The plots were smoothed with a 10-bp moving average. The base-pair-step parameters were taken from Liu et al., 2021 (Supplementary Table 2).

Sequence-based features including sequence-compositional information, DNA shape features, and DNA physical properties were calculated by merely using the DNA sequence as input. At first, we retrieved 1000-bp sequence for each hot/cold spot from the genome of *Saccharomyces cerevisiae* (SacCer3). Then, sequence-based features were calculated. GC-content, GC-skew, and MI were calculated along the sequence by using a sliding window of 100, 100, and 200 bp, respectively. K-mer composition was calculated for central 300-bp (or 150- and 500-bp) regions of the sequences. Other sequence-based parameters (DNA shape features and DNA physical properties) were calculated at each base-pair step and smoothed with a 10-bp average. Based on these data, distribution profile plots for the features (e.g., Figures 2–4) were generated. Finally, mean and variance of the sequence-based parameters along the central 300 bp were calculated and used as final features in the prediction. Calculated variance hear measures the variation of sequence-based parameters along the sequence. Utilizing the processed data available online, non-DNA features were calculated by averaging the signals within 300 bp regions at hot/cold spots. Variance was not calculated for non-DNA features.

### Classifiers

#### Random Forest

Random Forest (RF) is one of the widely used ensemble learning algorithms (Breiman, 2001). It generates numerous decision trees based on the training set and then majority voting strategy is used to label the class of the sequences in the test set. Its success in various fields is ascribed partially to de-correlating the bootstrap sampling decision trees by random sampling sub-sized features from the whole feature space at each splitting node. A RF-based model was developed to classify recombination hot/cold spots by using R package “randomForest”. To be specific, after the benchmark dataset was prepared, we characterized each sequence and prepared feature matrix for benchmark dataset. The number of features sampled from the feature space at each splitting point was set to log_2_*m* where *m* is total number of features in feature space. Optimal number of decision trees generated in the RF was set to 130 by inspecting Error-tree plot. Five-fold cross-validation was performed to evaluate the model.

#### Support Vector Machine

Support vector machine (Cortes and Vapnik, 1995) is an efficient classifier which has been widely used to solve classification and regression tasks. In SVM algorithm, input data (feature data) is mapped to a new feature space with higher dimension by using a kernel function and then optimal separating hyperplane is determined in the new feature space. In the current study, linear kernel was used to implement SVM-based classification using R package “e1071” with default values for all other parameters.

#### Logistic Regression

Logistic regression model is a generalized linear model that is used to predict the probability of a binary (yes/no) event occurring based on a set of independent variables (Collins et al., 2004; Nick and Campbell, 2007). In brief, the model the outcome of multiple regression is mapped to logistic function (sigmoid function), which is then transformed to eq. (5) by logit transform and the result of a binary event is predicted based on a threshold value (e.g., 0.5). In our model, independent variables are sample features, and the dependent variable is the label of the sample (e.g., hotspot or coldspot). The regression coefficients are estimated based on train dataset, and the outcomes of test samples are predicted.

(5) $$\mathrm{logit}\left(p\right)=\ln\frac{p}{1-p}=\theta_{0}+\theta_{1}x_{1}+\theta_{2}x_{2}+\ldots +\theta_{n}x_{n}$$

#### Naive Bayesian Classifier

Naive Bayesian classifier is a simple and fast classification algorithm (Friedman et al., 1997), which has been successfully used for many machine learning purposes and works particularly well in text classification. It uses Bayes’ Theorem to predict the label of a sample. “Naive” means the assumption that the occurrence of features is independent with each other, and thus likelihood *P*(*x*|*c*) is calculated as the product of each feature’s likelihood *P*(*x*_*i*_|*c*) as indicated in eq. (6). Likelihood probability for each feature is estimated by a Gaussian model. Then two posterior probabilities are calculated for each test sample by using Bayes theorem and the larger probability indicates the class (label) of the sample.

(6) $$P\left(c|x\right)=\frac{P\left(c\right)P\left(x|c\right)}{P\left(x\right)}=\frac{P\left(c\right)}{P\left(x\right)}\prod_{i}P\left(x_{i}|c\right)$$

Where*P*(*c*|*x*)is posterior probability that represents the probability of observing class *c* (*c* = hotspot or coldspot) given feature set *x*, *P*(*c*) is prior probability, and *P*(*x*|*c*) is class-conditional probability (likelihood).

#### Decision Tree

Decision tree describes the classification process of samples based on features (Quinlan, 1986). In other words, it consists of a series of decision rules that divide samples contained in each node into two or more subsets according to a feature-based decision rule. Decision tree begins with a root node representing training samples, and recursively generates new branches and nodes by using feature-based “if-then” rule until the node cannot be further classified. The final nodes are called leaf nodes. At each decision step, the best feature is used. Best feature for each node (root node or internal decision node) can be selected by a quantitative measurement method such as Gini index or Information Gain. Based on training data-based decision tree, the labels of test samples are predicted. The typical algorithm of decision tree is CART (Breiman et al., 1984), and we used R package “rpart” to develop CART-based decision tree classifier (parameters used in rpart function: method = “class,” cp = 0.000001).

### Assessment of Model Performance

Five-fold cross-validation was performed for each of the five classifiers introduced above, and overall performances were reported. The performance of classification model is quantified by widely used metrics including Sensitivity (*SN*), Specificity (*SP*), Accuracy (*ACC*), *F-measure*, and Area Under ROC curve (AUC)

(7) $$SN=\frac{TP}{TP+FN}$$

(8) $$SP=\frac{TN}{TN+FP}$$

(9) $$ACC=\frac{TP+TN}{TP+FP+TN+FN}$$

(10) $$F-\mathrm{measure}=\frac{2\times \text{precision}\times \text{recall}}{\text{precision}+\text{recall}}=\frac{2TP}{2TP+FN+FP}$$

where *TP*, *FN*, *TN*, and *FP* denote, respectively, the numbers of true positive, false negative, true negative, and false positive samples. *F-measure* is the harmonic mean of the precision and recall.

## Results and Discussion

### Characterization of Hotspots

To show how DNA-based features distribute at hot/cold spots, we plotted the average profile of DNA-based parameters at hot/cold spots (Figures 2–4). It is apparent that some of the parameters exhibit a clear characteristic pattern at hotspots, contrasting with random distributions at coldspots. For example, GC-skew shows a characteristic reversed skew between the two sides of the hotspot center, probably due to mutational bias (Smagulova et al., 2011); Mutual information has a dramatic peak at hotspot center (Figure 2), suggesting the possible biased usage of dinucleotides (Liu and Li, 2008); DNA shape parameters such as slide, shift, rise, helical twist, roll, stretch, opening, propeller twist, and minor groove width (MGW) show a peak or dip at the hotspot center (Figure 3). The force constants reflecting the deformation rigidity with regard to corresponding degrees of freedom also differ between hotspots and coldspots (Figure 4). It is worth noting that some of the distribution patterns of base-pair step parameters calculated based on our previously estimated parameter set (Figure 4) differ from DNAshapeR-based results (Figure 3). For example, both tilt and shift exhibit an anti-symmetric pattern with respect to hotspot center in Figure 4, while this pattern is absent for DNAshapeR-based results (Figure 3). It would be interesting if the specific patterns observed in Figure 4 represent an intrinsic feature of recombination hotspots. We also presented the distribution patterns of some other DNA physical properties at hot/cold spots (Supplementary Figure 3).

**FIGURE 5:**
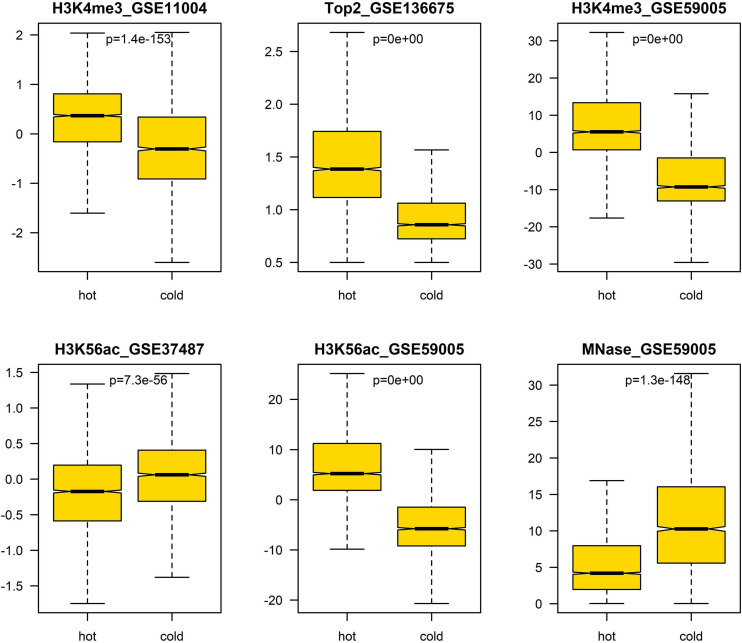
Difference of epigenetic marks between hot/cold spots. The *p*-values were obtained via *t*-test.

In addition, we also analyzed the difference of several epigenetic signals between hotspots and coldspots (Figure 5). The results show that H3K4me3, H3K56ac, MNase-seq signal, and Top2 binding signal differ between hotspots and coldspots. High levels of H3K4me3 and H3K56ac and reduced MNase-seq signal at hotspot center are usually used to indicate high chromatin accessibility. The enrichment of top2 binding at hotspots was reported previously (Gittens et al., 2019). It is unexpected that two H3K56ac datasets show different enrichment patterns (Figure 5), and the reason for the discrepancy is unclear.

### Performances of Classification Models

#### DNA-Based Prediction

We first focus on DNA-based prediction as many others done before. DNA-based features can be divided into two types: DNA compositional features and DNA physical properties. Let’s start with DNA compositional features.

**FIGURE 6:**
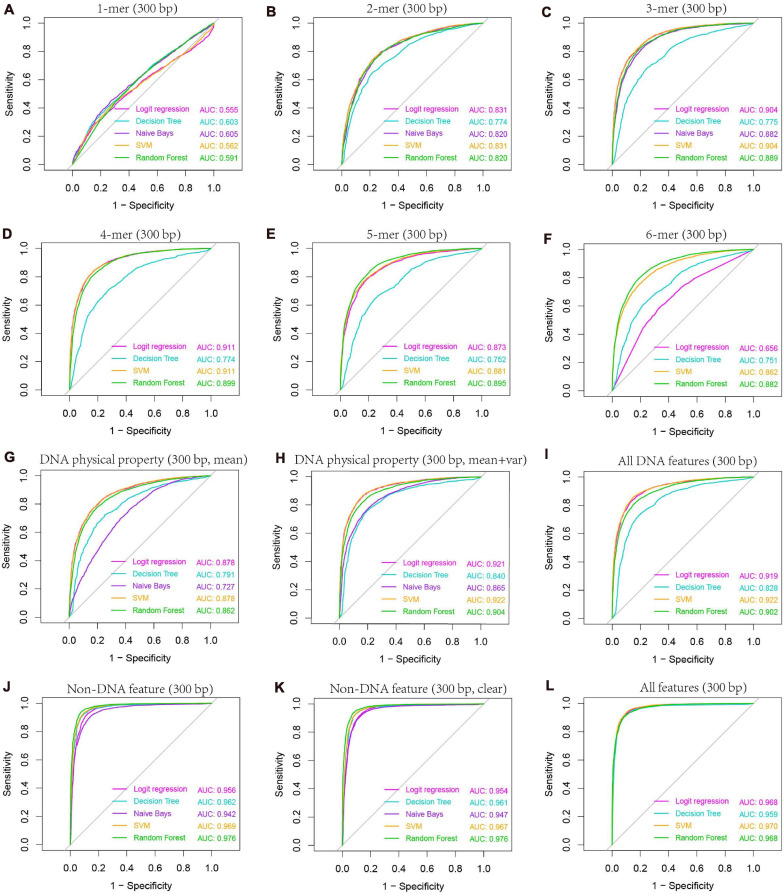
AUC-based comparison of prediction performance between different classification models. Results are based on combined decision values inferred from five-fold cross validation. Features including k-mer composition **(A–F)**, DNA physical properties **(G,H)**, and several non-DNA features (H3K4me3, H3K56ac, MNase-seq signal, and Top2 binding signal) were obtained from 300-bp regions centered at hot/cold spots. Mean and variance were calculated for DNA physical property features by averaging across the 300-bp genomic regions for each hot/cold spot. In non-DNA features **(J)**, predictions were based on six features (H3K4me3_GSE11004, H3K4me3_GSE59005, H3K56ac_GSE37487, H3K56ac_GSE59005, MNase_GSE59005, and Top2_GSE136675), and those excluding two redundant features (H3K4me3_GSE11004 and H3K56ac_GSE37487) were denoted as “clear” **(K)**. All DNA features **(I)** include 4-mer composition, GC-content, GC-skew, mutual information, DNA physical property features listed in Table 1. Note that DNA physical property features here include DNA physical properties and DNA shape parameters. All features include all DNA features and clear non-DNA features **(L)**.

Our previous study as well as others’ show that k-mer composition is related to recombination hotspots (Liu et al., 2012). To gain knowledge about which size of k-mer (*k* = 1–6) has the best predictive ability to discriminate hotspots from coldspots, we trained classifiers on k-mer probability features, where *k* ranges from 1 to 6, and predicted the class of test set samples. Our results based on five-fold cross-validation show that 4-mer composition is the best predictor (Supplementary Table 5 and Figure 6), achieving an accuracy of ∼83.7% by SVM-based classifier. Among the five classifiers, SVM performs the best, followed by logistic regression and RF. Naive Bays classifier is unstable when *k* is larger than four, which might be caused by inadequate sampling of k-mers in short sequences (300-bp) we used. Because many k-mers when *k* is 4–6 have zero occurrence in a short DNA sequence, and the derived probability of zero for the k-mers does not represent true case. Even if we introduced pseudo-count to smooth the k-mer probability, Naive Bays classifier still performs badly. Particularly for Naive Bays classifier, Gaussian distribution-based maximum likelihood estimate of posterior probability is unreliable, or even un-computable, because many zero values of k-mer occurrence (or homogeneous value of smoothed probability) may result in the variance of zero for a particular k-mer feature in feature space (4^*k*^ features), making the Gaussian probability density used in maximum likelihood estimate of posterior probability un-computable. In addition, predictions based on sequences shorter or longer than 300 bp (e.g., 150 and 500-bp) could not generate improved accuracy, suggesting that 300 bp is a proper window size for hotspot prediction (Supplementary Table 5).

**FIGURE 7:**
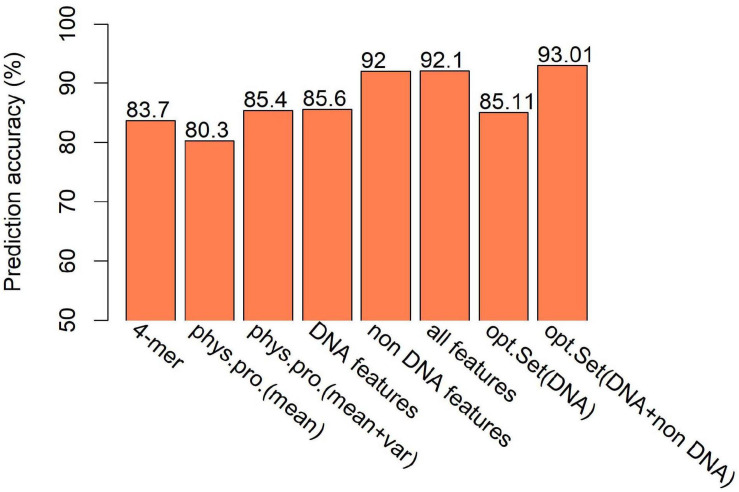
Comparison of SVM-based prediction accuracy between various feature sets. Feature range used in the prediction is 300 bp. Results are based on combined decision values inferred from five-fold cross validation. “phys.pro.” denotes physical property-based prediction, and “opt.Set” denotes optimal feature set.

The second class of DNA-based features is DNA physical properties, which impact DNA deformation such as DNA bending, stretching, base-paring and stacking. DNA shape parameters were included in this category. When predicting hot/cold spots based on this feature set, a worse prediction accuracy (Supplementary Table 6, SVM: ACC = 80.3%) than the 4-mer compositional features (Supplementary Table 5, SVM: ACC = 83.7%) was obtained (Figure 7). Again, predictions based on 300-bp window-based feature extraction are better than 150- and 500-bp window (Supplementary Table 6).

We then ask if the variation of sequence-based parameters along the sequences (see Figures 2–4) contributes to hot/cold spot classification. To test this, we included the variance of the sequence-based features along the sequences in feature set, and made predictions. The results show that the variation of the parameters indeed remarkably improved the prediction performance (Supplementary Table 7 and Figure 7, ACC = 85.4 vs. 80.3%). Combination of all the DNA-based features produced a prediction accuracy of 85.6% (Figure 7 and Supplementary Table 8).

#### Non-DNA Features Are a Strong Predictor of Recombination Hotspots

After evaluating the influence of DNA sequence information on discriminating hotspots and coldspots, we then sought to uncover how non-DNA features affect the identification of hotspots. Based on prior knowledge discovered in other experimental studies, we considered several types of non-DNA features: MNase-seq signal, histone modification signals (H3K4me3 and H3K56ac), and Top2 signal. It is apparent that this non-DNA feature set is capable of classifying hot/cold spots with a much higher accuracy (Figure 6J, AUC = 0.969) than DNA sequence-based features (Figure 6I, AUC = 0.922). It is unexpected that H3K56ac signal difference between hotspots and coldspots differs between two independent studies from which we obtained H3K56ac data (Figure 5). But in both studies (Hu et al., 2015; Karányi et al., 2020), H3K56ac was claimed to have positive contribution to recombination, probably due to H3K56ac-promoted chromatin accessibility which favors the binding of recombination machinery to hotspots. We therefore carried on prediction after removing the unexpected H3K56ac feature (H3K56ac_GSE37487) as well as one of redundant H3K4me3 features (H3K4me3_GSE11004) from our feature space. We see that even based on the only four non-DNA features, we still obtained high prediction accuracy (Figure 6K and Supplementary Table 9). Non-DNA features obtained from 150-bp regions led to almost the same prediction accuracy than features based on 300-bp span (Supplementary Table 9).

It is interesting that among the five classifiers used in this study, RF performs best when using non-DNA features, but SVM is the best when prediction is based on DNA features (Supplementary Table 9). This suggests that prediction performance is determined by the combinatorial effect of features and classification algorithm. Overall, SVM works the best with the whole feature set which consists of DNA-based features and non DNA features (Supplementary Table 10). The feature matrices for hot/cold spots were available at https://github.com/gqliu1010/Rec_hotspots.

#### Effect of Hot/Cold Spot Length on Prediction Performance

**FIGURE 8:**
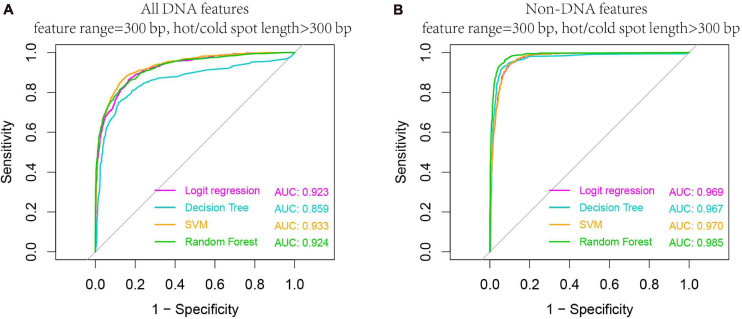
Higher AUC values are obtained when predicting larger hotspots (>300 bp). Results are based on combined decision values inferred from five-fold cross validation by using all DNA features **(A)** and non-DNA features **(B)**. See Table 1 for feature details.

We carried out our prediction above on the whole hot/cold spots dataset by calculating features from equally sized regions (e.g., 300-bp regions), without considering the potential effect of hot/cold spot length. Given the variable size of hot/cold spots, it is conceivable that features are also size-dependent. To investigate size-related effect, we selected the hot/cold spots that are larger than 300 bp, and re-examined if prediction accuracy is affected in this case. Our results show that both DNA-based and non-DNA feature-based prediction got increased accuracy (Figure 8 vs. Figure 6), indicating that longer hot/cold spots are more predictable as their underlying DNA sequence and epigenetic information are more informative than shorter hot/cold spots.

#### Comparison With Existing Models

In order to assess the performance of models presented in this study, we compared with some other existing computational models designed to predict hot/cold spots. Hold-out validation is used for prediction: randomly sampled 70% of the whole benchmark dataset is used to train models and the remaining 30% is used as test set. All the compared models made predictions on the same test set. As far as we know, previously developed models for recombination hot/cold spot classification are all based on DNA-based features. Hence, in order to make comparison more objective, we compared our DNA-based models with existing models.

The results show that our model achieved similar level of prediction accuracy (Table 2, SVM: ACC = 85.1%) as aforementioned five-fold cross-validation (Supplementary Table 8, SVM: ACC = 85.6%). However, applying the webservers for two other start-of-art models to the same test dataset, we obtained prediction accuracy of ∼60%, which is worse than our models. Why do the start-of-art models have so poor power to discriminate hot/cold spots? It is most likely because those models were trained on ORF sequences with high DSB frequency, while hotspots and coldspots in this study were rigorously defined based on high resolution Spo11 oligo-seq data. Although it was reported that recombination hotspots in budding yeast prefer promoter regions and may have overlap with coding region (Mancera et al., 2008), it is inappropriate to represent a hotspot with its adjacent ORF as coding regions and non-coding genomic regions differ a lot in terms of composition, structure and function. Thus, ORF-based training is not the best choice in computational models and may fail to predict rigorously defined hot/cold spots. Indeed, an IDQD model (Liu et al., 2012) trained on the hot/cold spots defined in this study achieved a much successful prediction (Table 2).

**TABLE 2:** The performances of several models in discriminating recombination hot/cold spots (feature range = 300 bp).

Method	Feature	*SN* (%)	*SP* (%)	*TA* (%)	*F-measure*
iRSpot-PseDNC^a^	PseDNC	47.3	56.9	51.3	53.2
iRecSpot-EF^b^	DNA-based features	38.8	71.5	51.8	49.3
IDQD	4-mer	82.8	83.3	83.0	85.1
SVM (current study)	All DNA features	85.1	85.0	85.1	86.8
RF (current study)	All DNA features	87.0	79.0	83.6	86.0
Logistic regression (current study)	All DNA features	86.2	81.1	84.0	86.2

*^*a*^Prediction from Chen et al., 2013. ^*b*^Prediction from Jani et al., 2018.*

#### Feature Importance and Optimal Feature Set

**FIGURE 9:**
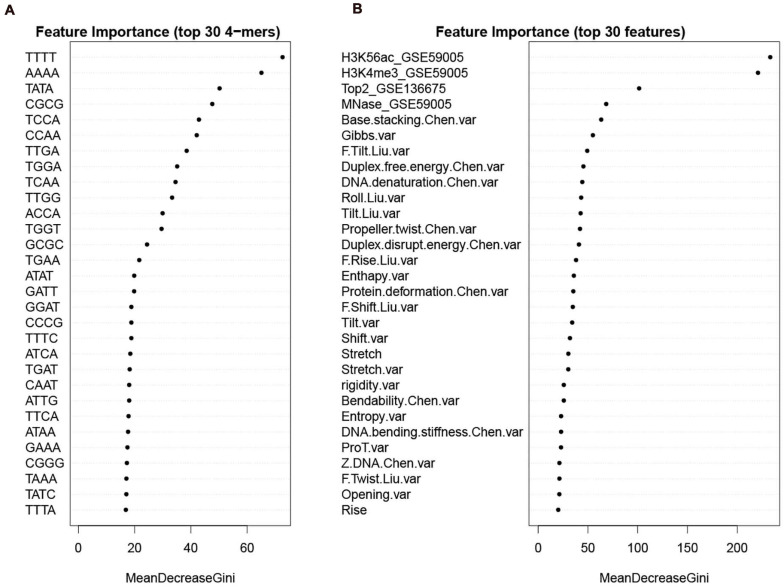
Feature importance sorted by Gini index. **(A)** top 30 k-mers; **(B)** top 30 features selected from the whole feature set. Examples of feature name illustration: “F.Slide.Liu.var” represents the variance of force constant for slide taken from Liu et al. (2021); “Duplex.free.energy.Chen.var” represents the variance of “Duplex free energy” taken from Chen et al. (2012); “Stretch” is DNA shape parameter calculated by using DNAshapeR. See Supplementary Tables 2–4 for more details about the features.

To give information about what features weigh much in our computational model, we first sorted the features according to Gini index that has been widely used to measure feature importance. The feature importance was inferred from the RF model trained on the whole benchmark dataset. We see that in DNA features, the variations of the DNA-based parameters along sequences rank high and composed the majority of the top 30 features (Figure 9B). Stretch and mutual information rank in the top 30. In addition, the list of top 30 4-mers (Figure 9A) indicates that oligomers such as AAAA/TTTT, TATA, and CGCG are important in hot/cold spot classification.

**FIGURE 10:**
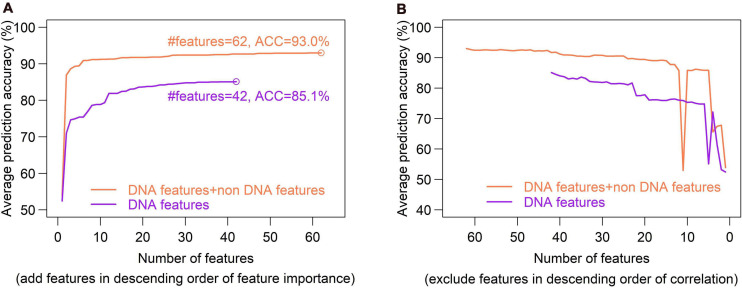
Optimal set of DNA features is determined through a SVM-based Incremental Feature Selection method (IFS). **(A)**, In the IFS method, ANOVA was used to sort feature importance and then features were added one by one to feature space in the descending order of feature importance. For each turn of feature addition, SVM classifier was trained on the updated feature set, and average prediction accuracy of five-fold cross validation was reported. If the addition of a feature increases the average prediction accuracy, the feature was retained in the feature set, otherwise it was removed. Optimal sets were sought, respectively, in DNA-based feature space (DNA features) and the whole feature space (DNA features + non DNA features). Two optimal feature sets composed of 45 features and 44 features were obtained. **(B)**, Inter-correlated features were excluded sequentially from the feature sets obtained in figure **(A)**. During the feature-excluding process, no new peak was observed for prediction accuracy, and thus the optimal feature set determined through IFS remain unchanged.

Feature selection is crucial in machine learning, because the high dimension of feature space often cause high risk of over-fitting and make the prediction model computationally expensive. There are several feature selection strategies, such as filter, wrapper and embedding. We used IFS method (Zhang et al., 2021), which is a filter-based approach, to obtain an optimal feature set which can give best prediction. In the IFS method, analysis of variance (ANOVA) was used to assess feature importance. The features were sorted according to the decreasing order of the ratio between inter-group variance and intra-group variance. The higher the ratio is, the more powerful the feature is in discriminating the two groups of samples (hotspots and coldspots). Then the features were added one by one to feature space in the descending order of feature importance. For each turn of feature addition, SVM classifier was trained by using the new feature set, and average prediction accuracy of five-fold cross validation was reported (Figure 10A). If the addition of a feature increases the average prediction accuracy, the feature was retained in the feature set, otherwise it was removed. Optimal sets were sought, respectively, in DNA-based feature space and the whole feature space. We show that our model based on the optimal feature set which consists of only 62 features achieved a slightly improved accuracy than all-feature-based model (Figure 7, 93 vs. 92.1%). In addition, we also examined the overlap between top 50 features determined, respectively, by Gini index and ANOVA. Most of them (80%) occur in both feature sets, suggesting the consistency of feature importance between the two methods (Supplementary Figure 4). The consistent features occurred in both top feature sets might represent the most important features (Supplementary Table 11).

Excluding redundant features is another way to reduce feature dimensionality with no or little sacrifice in prediction accuracy. If two features strongly correlate with each other, it is possible that only one of them is sufficient for prediction. We used a recursive redundant-feature-excluding method, in which highly correlated features are excluded one by one from the optimal feature set according to the descending order of Pearson’s correlation coefficients between features. One of the two correlated features, performing worse in univariate classification, was removed at each round, and then the model was re-trained on the updated feature set of training dataset, followed by a five-fold cross validation. The univariate classification means individual feature-based classification. During the feature-excluding process, no new peak was observed for prediction accuracy, and thus the optimal feature set determined through IFS remained unchanged (Figure 10B). We can also see that the earliest removal of features which represent the exclusion of highly correlated (redundant) features has little impact on prediction accuracy, while the later-removal of features affect prediction accuracy remarkably (Figure 10B).

## Conclusion

In summary, firstly we defined a reliable set of recombination cold spots based on high-resolution Spo11-oligo sequencing data; secondly, we characterized recombination hot/cold spots in terms of sequence-derived features and epigenetic marks; thirdly, we performed binary predictions based on five classification algorithms. Our results show that, overall, SVM classifier performs the best in hot/cold spot classification, and also outperforms other existing methods. Importantly, our results indicate that variance in sequence-based feature profile and epigenetic marks are able to assist remarkably the identification of recombination hotspots.

## Data Availability Statement

The original contributions presented in the study are included in the article/Supplementary Material, further inquiries can be directed to the corresponding author/s.

## Author Contributions

GQL developed the model, carried out the analysis, and wrote the manuscript. SS and YS carried out the partial calculation of DNAshape parameters. SS, QZ, BD, YS, GJL, and XZ participated in the data analysis and discussion. All authors contributed to the article and approved the submitted version.

## Conflict of Interest

The authors declare that the research was conducted in the absence of any commercial or financial relationships that could be construed as a potential conflict of interest.
