# Supplementary material for: Epigenetic Marks and Variation of Sequence-Based Information Along Genomic Regions Are Predictive of Recombination Hot/Cold Spots in Saccharomyces cerevisiae
Source: Front Genet. 2021 Jun 29;12:705038. doi: 10.3389/fgene.2021.705038 (PMC8276760; doi:10.3389/fgene.2021.705038)
Supplement: Supplementary file 1 [file Data_Sheet_1.PDF]

Supplementary Materials

"Epigenetic marks and variation of sequence-based information along genomic regions are predictive of recombination hot/cold spots in Saccharomyces cerevisiae"

"

Guoqing Liu, Shuangjian Song, Qiguo Zhang, Biyu Dong, Yu Sun, Guojun Liu, Xiujuan Zhao

Figure S1. The length distribution of recombination hotspots and coldspots.

Figure S2. Schematic illustration of DNA base-pair step parameters.

Figure S3. Distribution of DNA physical properties at hot/cold spots. The plots were smoothed with a 10-bp moving average. The physical property parameters were taken from Chen et al. 2011 (Table S3) and Ignatova et al. 2008 (Table S4).

Figure S4. Venn diagram for top 50 features determined respectively by Gini index and ANOVA.

Table S1. The recombination hotspots and coldspots used in this study (provided as a separate EXCEL file)

Table S2. Force constants and equilibrium structure parameters for base-pair steps taken from Liu et al., 2021

Table S3. DNA physical parameters for base-pair steps collected in Chen et al., 2012

Table S4. DNA physical parameters for base-pair steps taken from Ignatova et al., 2008

Table S5. The performances of k-mer composition-based classifiers in discriminating recombination hot/cold spots

Table S6. The performances of physical property-based classifiers in discriminating recombination hot/cold spots (mean)

Table S7. The performances of physical property-based classifiers in discriminating recombination hot/cold spots (mean+variance)

Table S8. The performances of all-DNA-feature-based classifiers in discriminating recombination hot/cold spots

Table S9. The performances of non-DNA-feature-based classifiers in discriminating recombination hot/cold spots (clear)

Table S10. The performances of all-feature-based classifiers in discriminating recombination hot/cold spots

Table S11. The list of features overlapped between top 50 features determined respectively by Gini index and ANOVA

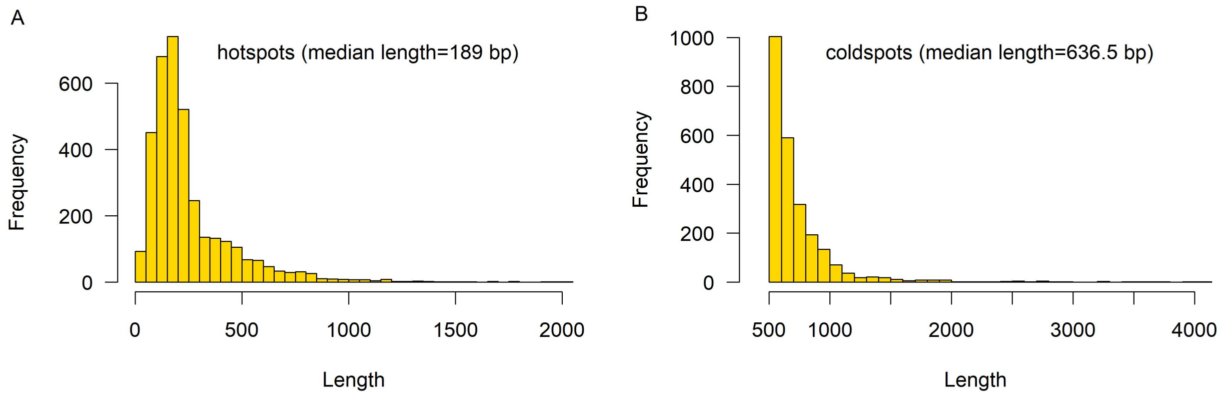

Figure S1. The length distribution of recombination hotspots and coldspots.

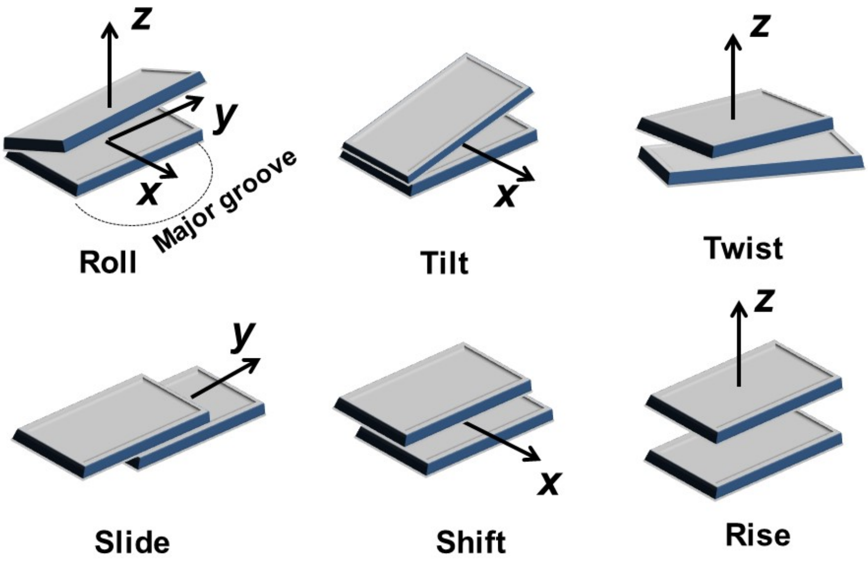

Figure S2. Schematic illustration of DNA base-pair step parameters.

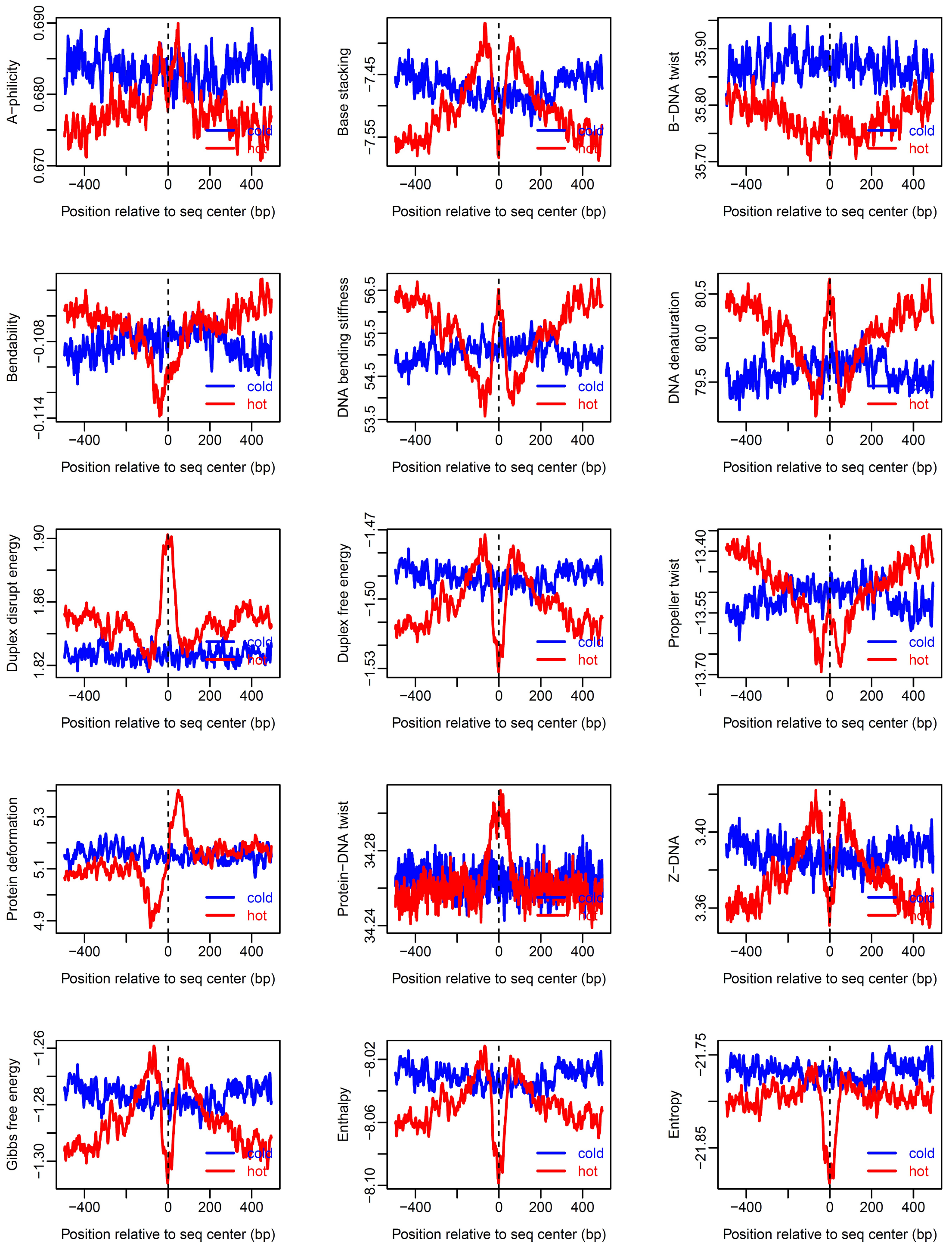

Figure S3. Distribution of DNA physical properties at hot/cold spots. The plots were smoothed with a 10-bp moving average. The physical property parameters were taken from Chen et al. 2011 (Table S3) and Ignatova et al. 2008 (Table S4).

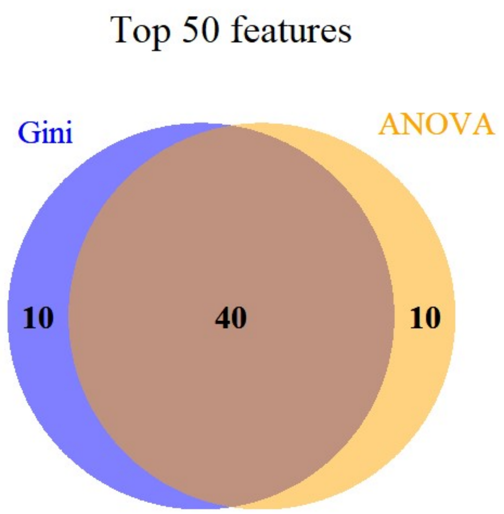

Figure S4. Venn diagram for top 50 features determined respectively by Gini index and ANOVA.

Table S2. Force constants and equilibrium structure parameters for base-pair steps taken from Liu et al. 2021

	Force constants						Equilibrium parameters					
step	k(Shift)	k(Slide)	k(Rise)	k(Tilt)	k(Roll)	k(Twist)	Shift	Slide	Rise	Tilt	Roll	Twist
AA	6.641	6.845	26.514	0.113	0.041	0.071	-0.001	-0.176	3.253	-1.365	1.049	35.031
AT	4.65	9.583	24.819	0.098	0.054	0.07	0	-0.679	3.208	0	0.612	30.726
AG	3.393	3.501	21.303	0.086	0.044	0.056	0.151	-0.222	3.321	-1.52	3.538	32.259
AC	3.981	7.099	21.977	0.071	0.066	0.064	0.287	-0.594	3.243	0.275	2.015	31.524
TA	4.101	1.847	14.18	0.064	0.031	0.048	0	0.043	3.389	0	3.506	36.94
TT	6.641	6.845	26.514	0.113	0.041	0.071	0.001	-0.176	3.253	1.365	1.049	35.031
TG	3.143	2.018	14.642	0.057	0.035	0.052	0.162	0.482	3.366	0.182	5.607	35.404
TC	5.082	4.734	24.761	0.097	0.05	0.059	0.313	-0.037	3.299	1.58	2.473	35.743
GA	5.082	4.734	24.761	0.097	0.05	0.059	-0.313	-0.037	3.299	-1.58	2.473	35.743
GT	3.981	7.099	21.977	0.071	0.066	0.064	-0.287	-0.594	3.243	-0.275	2.015	31.524
GG	3.034	2.989	19.495	0.086	0.043	0.058	-0.079	-0.166	3.362	-0.784	4.705	33.537
GC	2.618	4.176	16.983	0.065	0.055	0.059	0	-0.192	3.267	0	1.743	34.06
CA	3.143	2.018	14.642	0.057	0.035	0.052	-0.162	0.482	3.366	-0.182	5.607	35.404
CT	3.393	3.501	21.303	0.086	0.044	0.056	-0.151	-0.222	3.321	1.52	3.538	32.259
CG	3.01	2.708	14.594	0.059	0.039	0.051	0	0.443	3.292	0	6.021	33.668
CC	3.034	2.989	19.495	0.086	0.043	0.058	0.079	-0.166	3.362	0.784	4.705	33.537

Table S3. DNA physical parameters for base-pair steps collected in Chen et al. 2012

step	A-philicity	Base stacking	B-DNA twist	Bendabili ty	DNA bending stiffness	DNA denaturation	Duplex disrupt energy	Duplex free energy	Propeller twist	Protein deformation	Protein-DNA twist	Z-DNA
AA/TT	0.97	-5.37	35.5	-0.27	35	66.51	1.9	-1.2	-18.66	12.1	35.1	3.9
AG/CT	0.33	-6.78	30.6	-0.08	60	85.12	1.6	-1.5	-14	6.3	31.9	3.4
AC/GT	0.13	-10.51	33.1	-0.21	60	108.8	1.3	-1.5	-13.1	9.8	31.5	4.6
AT	0.58	-6.57	43.2	-0.28	20	72.29	0.9	-0.9	-15.01	2.1	29.3	5.9
GA/TC	0.98	-9.81	39.6	0.03	60	80.03	1.6	-1.5	-13.48	2.3	36.3	3.4
GG/CC	0.19	-8.26	35.3	-0.06	130	99.31	3.1	-2.3	-8.11	6.1	32.9	2.4
GC	0.73	-14.6	38.4	0.02	85	135.83	3.1	-2.3	-11.08	4	33.6	4
GT/AC	0.13	-10.51	33.1	-0.18	60	108.8	1.3	-1.5	-13.1	2.1	31.5	4.6
CA/TG	1.04	-6.57	37.7	-0.01	60	64.92	1.9	-1.7	-9.45	6.1	37.3	1.3
CG	0.52	-9.69	31.3	-0.03	85	88.84	3.6	-2.8	-10.03	4.5	36.1	0.7
CC/GG	0.19	-8.26	35.3	-0.03	130	99.31	3.1	-2.3	-8.11	2.9	32.9	2.4
CT/AG	0.33	-6.78	30.6	-0.18	60	85.12	1.6	-1.5	-14	1.6	31.9	3.4
TA	0.73	-3.82	31.6	0.18	20	50.11	1.5	-0.9	-11.85	2.3	37.8	2.5
TG/CA	1.04	-6.57	37.7	0.13	60	64.92	1.9	-1.7	-9.45	9.8	37.3	1.3
TC/GA	0.98	-9.81	39.6	-0.11	60	80.03	1.6	-1.5	-13.48	4.5	36.3	3.4
TT/AA	0.97	-5.37	35.5	-0.28	35	66.51	1.9	-1.2	-18.66	2.8	35.1	3.9

Table S4. DNA physical parameters for base-pair steps taken from Ignatova et al. 2008

step	Gibbs free energy	Enthapy	Entropy
AA/TT	-1	-7.6	-21.3
AG/CT	-1.28	-7.8	-21
AC/GT	-1.44	-8.4	-22.4
AT	-0.88	-7.2	-20.4
GA/TC	-1.3	-8.2	-22.2
GG/CC	-1.84	-8	-19.9
GC	-2.24	-9.8	-24.4
GT/AC	-1.44	-8.4	-22.4
CA/TG	-1.45	-8.5	-22.7
CG	-2.17	-10.6	-27.2
CC/GG	-1.84	-8	-19.9
CT/AG	-1.28	-7.8	-21
TA	-0.58	-7.2	-21.3
TG/CA	-1.45	-8.5	-22.7
TC/GA	-1.3	-8.2	-22.2
TT/AA	-1	-7.6	-21.3

Table S5. The performances of k-mer composition-based classifiers in discriminating recombination hot/cold spots

classifier	feature	Feature region	*SN*	*SP*	*ACC*	*F-measure*
	1-mer	300-bp	0.986	0.000	0.579	0.733
	2-mer	300-bp	0.823	0.668	0.759	0.800
Logistic regression	3-mer	300-bp	0.862	0.776	0.826	0.853
	**4-mer**	**300-bp**	**0.859**	**0.805**	**0.837**	**0.861**
	5-mer	300-bp	0.820	0.761	0.795	0.825
	6-mer	300-bp	0.633	0.612	0.624	0.664
	1-mer	300-bp	0.581	0.274	0.454	0.555
	2-mer	300-bp	0.746	0.778	0.759	0.784
SVM	3-mer	300-bp	0.822	0.820	0.821	0.843
	**4-mer**	**300-bp**	**0.838**	**0.835**	**0.837**	**0.858**
	5-mer	300-bp	0.785	0.819	0.799	0.821
	6-mer	300-bp	0.746	0.816	0.775	0.796
	1-mer	300-bp	0.553	0.590	0.569	0.601
	2-mer	300-bp	0.741	0.772	0.754	0.779
Random Forest	3-mer	300-bp	0.816	0.808	0.813	0.837
	4-mer	300-bp	0.792	0.852	0.817	0.835
	5-mer	300-bp	0.845	0.786	0.821	0.847
	6-mer	300-bp	0.794	0.812	0.801	0.824
	1-mer	300-bp	0.604	0.538	0.577	0.626
	2-mer	300-bp	0.788	0.722	0.761	0.794
Naive Bays	3-mer	300-bp	0.829	0.778	0.808	0.835
	4-mer	300-bp	1.000	0.005	0.588	0.740
	5-mer	300-bp	0.999	0.002	0.587	0.739
	6-mer	300-bp	0.598	0.792	0.679	0.686
	1-mer	300-bp	0.394	0.764	0.547	0.505
	2-mer	300-bp	0.647	0.785	0.704	0.720
Decision Tree	3-mer	300-bp	0.684	0.751	0.712	0.736
	4-mer	300-bp	0.709	0.724	0.715	0.745
	5-mer	300-bp	0.642	0.760	0.691	0.709
	6-mer	300-bp	0.606	0.785	0.680	0.689
Logistic regression	4-mer	150-bp	0.838	0.768	0.809	0.837
SVM	4-mer	150-bp	0.796	0.839	0.814	0.834
Random Forest	4-mer	150-bp	0.788	0.804	0.795	0.818
Naive Bays	4-mer	150-bp	0.999	0.002	0.587	0.739
Decision Tree	4-mer	150-bp	0.664	0.755	0.702	0.723
Logistic regression	4-mer	500-bp	0.865	0.789	0.834	0.859
SVM	4-mer	500-bp	0.845	0.814	0.832	0.855
Random Forest	4-mer	500-bp	0.837	0.818	0.829	0.852
Naive Bays	4-mer	500-bp	1.000	0.006	0.589	0.740
Decision Tree	4-mer	500-bp	0.687	0.755	0.715	0.739

Table S6. The performances of physical property-based classifiers in discriminating recombination hot/cold spots (mean)

classifier	Feature range	*SN*	*SP*	*ACC*	*F-measure*
Logistic regression	150-bp	0.828	0.718	0.783	0.817
SVM	150-bp	0.718	0.836	0.767	0.783
Random Forest	150-bp	0.719	0.799	0.752	0.773
Naive Bays	150-bp	0.505	0.782	0.620	0.609
Decision Tree	150-bp	0.693	0.724	0.706	0.735
**Logistic regression**	**300-bp**	**0.843**	**0.753**	**0.806**	**0.836**
**SVM**	**300-bp**	**0.811**	**0.791**	**0.803**	**0.828**
Random Forest	300-bp	0.776	0.803	0.787	0.811
Naive Bays	300-bp	0.515	0.771	0.621	0.614
Decision Tree	300-bp	0.725	0.747	0.734	0.762
Logistic regression	500-bp	0.844	0.742	0.802	0.833
SVM	500-bp	0.801	0.801	0.801	0.825
Random Forest	500-bp	0.773	0.811	0.789	0.811
Naive Bays	500-bp	0.521	0.767	0.623	0.618
Decision Tree	500-bp	0.688	0.763	0.719	0.742

Table S7. The performances of physical property-based classifiers in discriminating recombination hot/cold spots (mean+variance)

classifier	Feature range	*SN*	*SP*	*ACC*	*F-measure*
Logistic regression	300-bp	0.877	0.815	0.851	0.874
**SVM**	**300-bp**	**0.866**	**0.838**	**0.854**	**0.874**
Random Forest	300-bp	0.854	0.804	0.833	0.857
Naive Bays	300-bp	0.689	0.869	0.763	0.773
Decision Tree	300-bp	0.755	0.810	0.778	0.800

Table S8. The performances of all-DNA-feature-based classifiers in discriminating recombination hot/cold spots

classifier	Feature range	*SN*	*SP*	*ACC*	*F-measure*
Logistic regression	300-bp	0.869	0.816	0.847	0.870
**SVM**	**300-bp**	**0.857**	**0.854**	**0.856**	**0.874**
Random Forest	300-bp	0.805	0.850	0.824	0.843
Naive Bays	300-bp	1.000	0.004	0.588	0.740
Decision Tree	300-bp	0.727	0.814	0.763	0.782

Note: all-DNA-feature set refers to all the DNA-based features (k-mer=4-mer) listed in Table 1.

Table S9. The performances of non-DNA-feature-based classifiers in discriminating recombination hot/cold spots (clear)

classifier	Feature range	*SN*	*SP*	*ACC*	*F-measure*
Logistic regression	150-bp	0.943	0.859	0.908	0.924
SVM	150-bp	0.929	0.910	0.921	0.933
Random Forest	150-bp	0.938	0.925	0.933	0.943
Naive Bays	150-bp	0.923	0.857	0.896	0.912
Decision Tree	150-bp	0.911	0.932	0.919	0.930
Logistic regression	300-bp	0.935	0.859	0.904	0.919
**SVM**	**300-bp**	**0.929**	**0.907**	**0.920**	**0.932**
**Random Forest**	**300-bp**	**0.939**	**0.921**	**0.932**	**0.942**
Naive Bays	300-bp	0.932	0.848	0.897	0.914
Decision Tree	300-bp	0.920	0.912	0.917	0.928

Note: all-feature set refers to the non-DNA features listed in Table 1, excluding two redundant features (H3K4me3_GSE11004, H3K56ac_GSE37487)

Table S10. The performances of all-feature-based classifiers in discriminating recombination hot/cold spots

classifier	Feature range	*SN*	*SP*	*ACC*	*F-measure*
Logistic regression	300-bp	0.930	0.895	0.916	0.928
**SVM**	**300-bp**	**0.942**	**0.893**	**0.921**	**0.934**
Random Forest	300-bp	0.905	0.914	0.908	0.921
Naive Bays	300-bp	1.000	0.003	0.588	0.740
Decision Tree	300-bp	0.921	0.902	0.913	0.926

Note: all-feature set refers to all the features (k-mer=4-mer) listed in Table 1, excluding two redundant features (H3K4me3_GSE11004, H3K56ac_GSE37487)

Table S11. The list of features overlapped between top 50 features determined respectively by Gini index and ANOVA

Feature Name
H3K56ac_GSE59005
H3K4me3_GSE59005
Top2_GSE136675
MNase_GSE59005
Base.stacking.Chen.var
Gibbs.var
F.Tilt.Liu.var
Duplex.free.energy.Chen.var
DNA.denaturation.Chen.var
Roll.Liu.var
Tilt.Liu.var
Propeller.twist.Chen.var
Duplex.disrupt.energy.Chen.var
F.Rise.Liu.var
Enthapy.var
Protein.deformation.Chen.var
F.Shift.Liu.var
Tilt.var
Shift.var
Stretch
Stretch.var
rigidity.var
Bendability.Chen.var
Entropy.var
DNA.bending.stiffness.Chen.var
ProT.var
Z.DNA.Chen.var
F.Twist.Liu.var
Opening.var
Rise
B.DNA.twist.Chen
Slide.Liu.var
F.Slide.Liu.var
HelT.var
Twist.Liu.var
Slide.var
GC.var
CGCG
Shift
A.philicity.Chen.var

References :

Chen, W., Lin, H., Feng, P. M., Ding, C., Zuo, Y. C., and Chou K. C. (2012). iNuc-PhysChem: a sequence-based predictor for identifying nucleosomes via physicochemical properties. PLoS One 7, e47843.

Ignatova, Z., Martinez-Perez, I., and Zimmermann, K. H. (2008). DNA Computing Models. New York: Springer

Liu, G., Zhao, H., Meng, H., Xing, Y., and Cai, L. (2021). A deformation energy model reveals sequence-dependent property of nucleosome positioning. Chromosoma, 130, 27-40.

Scipioni, A., Anselmi, C., Zuccheri, G., Samori, B., and De Santis, P. (2002). Sequence-dependent DNA curvature and flexibility from scanning force microscopy images. Biophys J, 83, 2408-2418.
